# Age-related asymmetry in anticipatory postural movements during unilateral arm movement and imagery

**DOI:** 10.1007/s00221-022-06416-5

**Published:** 2022-08-05

**Authors:** Chloe Wider, Suvobrata Mitra, Hayley Boulton, Mark Andrews

**Affiliations:** Department of Psychology, Nottingham Trent University, Nottingham, UK

**Keywords:** Posture control, Motor imagery, Aging, Anticipation, Anticipatory

## Abstract

Reaching movements of the arms are accompanied by anticipatory (APM) and compensatory postural motion (CPM) that counteract the resulting perturbations to body stability. Recent research has shown that these postural actions are also observable in the context of imagined arm movements. As motor imagery (MI) shares many neurophysiological and behavioral characteristics with physical movements, and MI training can affect subsequent performance, MI tasks provide a good setting for studying the anticipatory aspects of postural control. This study investigated APMs and CPMs of the head and hip of healthy young and older adults in the temporal vicinity of physical and imagined forward raises of the dominant and non-dominant arm. When MI of the dominant arm was self-initiated, both age groups showed APM in the anteroposterior plane. When the self-initiated MI was of the non-dominant arm, only the older group showed anteroposterior APM. The older group did not show APM when an expected arm movement (or MI) was made to an external signal. This suggests an age-related deficit in coordinating postural preparation with external events. Only the older group showed mediolateral APM, and only for dominant arm MI, indicating sensitivity to potential perturbation to the weaker, non-dominant side of the body. Overall, the older group showed more anticipatory postural motion at the head. Systematic APM for manual MI suggests that MI training may be an effective intervention for anticipatory postural control. An integrated model of postural support for executed and imagined limb movements is suggested.

## Introduction

When humans extend their arms to perform goal-directed actions from an upright stance, or during gait, the posture control system must anticipate and counteract the resulting perturbation to the body’s balance (Massion 1992). The activation of leg muscles before the prime mover (Belenkiy et al. 1967), or the trunk’s backward bend to counteract the center of gravity’s (CG) forward motion (Martin 1967) are examples of anticipatory postural adjustments (APA) that are made in the direction opposite to the reaction forces produced by arm movement (Bouisset and Zattara 1987a, b, 1988; Cordo and Nashner 1982). A range of evidence suggests that one role of APAs is to regulate the CG (Bouisset and Zattara 1981, 1987b, 1988, 1990; Friedli et al. 1988; Mouchnino et al. 1990; Ramos and Stark 1990; Rogers and Pai 1990), and another may be to assist the arm movement by stabilizing its postural basis (Bleuse et al. 2006; Lee et al. 1990).

A close correspondence between APA and focal movement characteristics has been observed. For example, APA duration is longer when the arm lifts a greater load (Bouisset and Zattara 1988; Brown and Frank 1987; Zattara and Bouisset 1986). Also, APAs are affected separately by the magnitude of perturbation and the magnitude of action triggering the perturbation, which has led to APAs being considered an element of focal movement planning (Aruin and Latash 1995; 1996). APAs show significant adaptability of timing (Brown and Frank 1987; Cordo and Nashner 1982) and can occur even in the absence of a focal movement if a perturbation is expected (Aruin et al. 2001). More recently, postural movements have also been observed in the context of motor imagery (MI) of limb movements (Boulton and Mitra 2013, 2015; Grangeon et al. 2011; Rodrigues et al. 2010; Wider et al. 2020). Such results support the suggestion that APAs and their corresponding focal movements are planned and controlled through parallel central processes (Massion 1992).

The observation of postural motion in the temporal vicinity of manual MI is of particular interest because, in the absence of physical focal movement, postural adjustments during MI likely reflect the posture control system’s response to the expected (but not delivered) postural perturbation the focal movement would generate. A wide range of evidence (Guillot and Collet 2010) shows that MI activates cortical mechanisms (Bonnet et al. 1997; Clark et al.^2004^; De Lange et al. 2006; Grèzes and Decety 2001; Orr et al. 2008) and corticospinal (Stinear et al. 2006) pathways as well as the motor periphery (De Lange et al. 2006; Guillot et al. 2007; Kaneko et al. 2003; Lebon et al. 2008; Vargas 2004). MI also generates the patterns of autonomic arousal that are associated with motor planning (Collet et al. 2013). So, postural adjustments during MI may provide useful information about the feedforward aspects of the postural component of motor planning. This is of particular interest in the context of apparently greater dependence of motor control on execution-time visual feedback in older people (Haaland et al. 1993). Aging is also associated with sensorimotor attenuation, whereby the perceived intensity of sensory signals from self-generated as opposed to external actions reduces. This adds to the load of integrating sensory information with predictions of the results of action made by internal models (Wolpe et al. 2016). Thus, anticipatory postural action becomes an increasingly important process in older age.

In the context of MI, Wider et al. (2020) was the first to show that anticipatory postural motion occurred during imagined movements. They investigated whether the postural motion of standing young (Y) and older (O) adults’ head and hip within the time windows of 1000 ms before and after the onset of physical and imagined arm movements had distinguishable anticipatory and compensatory characteristics, respectively. They chose a 1000 ms anticipatory period to pick up the effects of both the early (preparatory) and the anticipatory postural activity that have been distinguished in previous research (Krishnan et al. 2012; Lee et al. 1990). Their participants performed physical and imagined arm raises under self-initiated and externally triggered task conditions. Under self-initiated conditions, only O showed anticipatory postural motion just before physical arm raises, whereas both O and Y showed anticipatory postural motion just before imagined arm raises. Under externally triggered conditions, Y showed anticipatory postural motion just before physical and imagined arm raises, but O did not show anticipation in either task condition. Wider et al.’s (2020) results showed that anticipatory postural motion does precede manual MI but this process is attenuated in O when they do not have control over the timing of movement onset.

Wider et al. (2020) studied symmetrical, bilateral arm raises and reported only on anteroposterior (AP) postural motion. Postural adjustment in the case of unilateral arm movement is arguably even more important with respect to the impact of ageing because such manual movements are more likely to perturb posture mediolaterally. It is well known that ageing particularly affects mediolateral (ML) postural stability (Brauer et al. 2000; Maki et al. 1994; Pasma et al. 2014) and the ability to counteract perturbations in the ML direction (Claudino et al 2013; Scariot et al. 2016). Research also suggests that O are more prone to asymmetry in body weight distribution, particularly in the absence of vision (Blaszczyk et al. 2000), though this was not specific to either the dominant or non-dominant side of the body. It is thought that this is a compensatory strategy and that O shift body weight so that the stronger limb is ready to take a recovery step in case of perturbation. Along these lines, Skelton (2002) found that asymmetric muscle power in legs is more pronounced in O with a history of falls;, however, asymmetry in fallers did not correlate with the reported number of falls.

There is also evidence that movements of the dominant and non-dominant arm differ in the postural adjustments they elicit. For example, Huang (2009) reported that Y and O’s APA amplitude and duration were greater for reaches of the dominant arm. Teyssèdre et al (2000) showed that the asymmetry of postural adjustment during dominant and non-dominant arm movement depends on the body’s level of stability. When seated in a stable position, APAs started earlier and arm velocity was higher for the dominant arm. However, when the seating position was less stable, arm velocities did not differ but greater postural muscle activity occurred for movements of the non-dominant arm. Evidence also suggests that the trajectories followed by reaching movements of the dominant and non-dominant arm can have different kinematic and kinetic (Bagesteiro and Sainburg 2002) characteristics. Reaching movements of the dominant arm have less variable mediolateral curvature than those of the non-dominant arm (Sainburg and Kalakanis 2000). Studies of the torque patterns and corresponding EMG profiles also show that dominant movements are more torque efficient. These factors are likely to affect the perturbation that the postural adjustments must anticipate and counteract.

## The present study

**Fig. 1:**
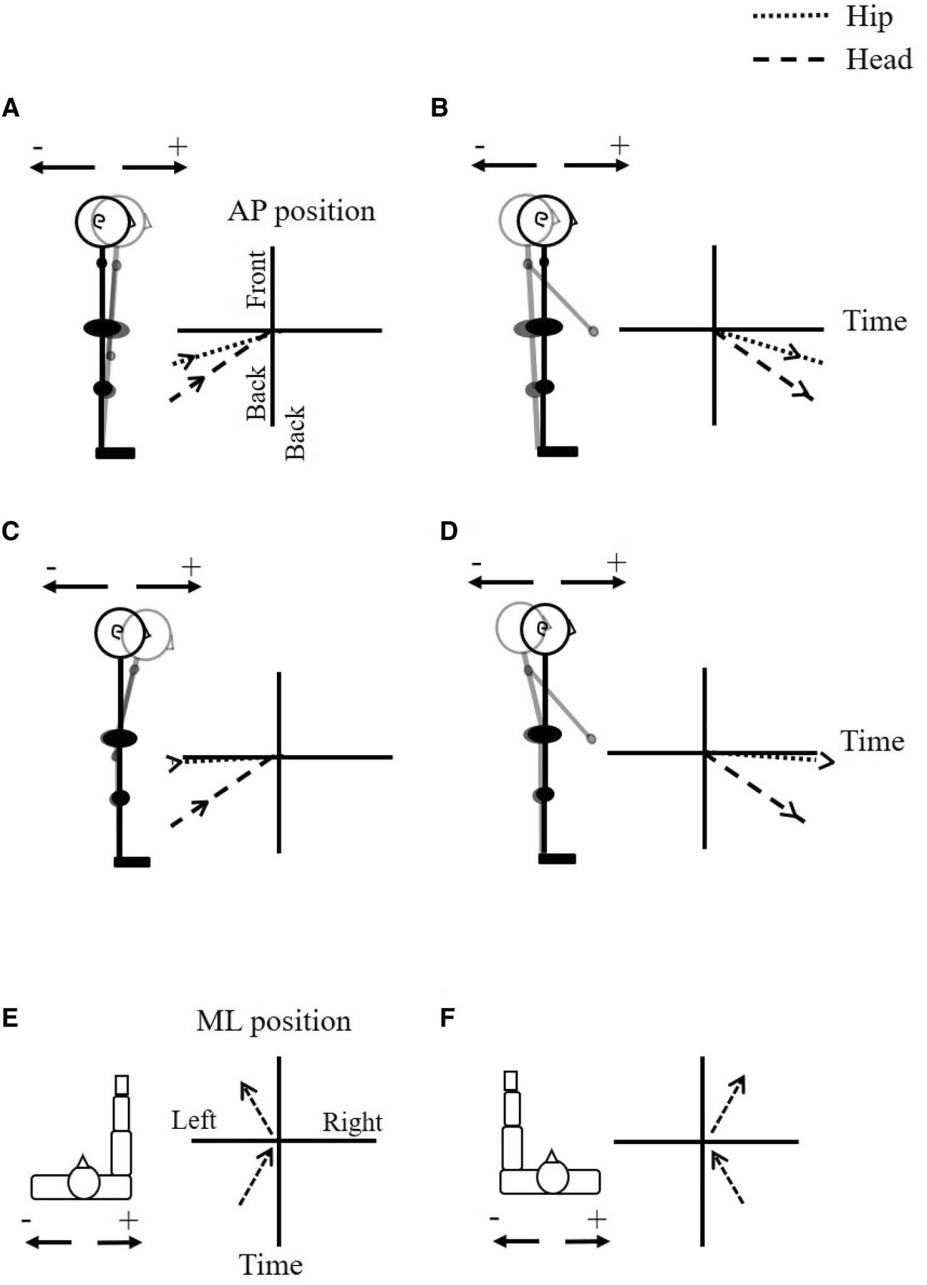
Representation of anticipatory and compensatory postural motion. Panels **A**–**D** show anteroposterior postural motion, where the vertical axis represents anteroposterior (AP) hip or head position and the horizontal axis represents time. The origin is at the onset of arm movement (or imagery). The time and hip and head positions at this moment are set to zero. Figures 2 and 4 use the convention shown in panels (**A**–**D**). Panel **A** shows how forward motion made at the ankle joint would be represented, and panel **B** shows the corresponding backward motion. Panels **C** and **D** show the same movements made at the hip. For the forward arm raises studied here, anticipatory postural motion in the anteroposterior plane is expected to be in the forward direction (**A**, **C**), and compensatory postural motion in the backward direction (**B**, **D**). Panels **E** and **F** show the convention used to represent mediolateral postural motion (viewed from above). In this case, the vertical axis represents time, and the horizontal axis the mediolateral hip or head position. Figures 3 and 5 use this convention. When the movement (or imagery) is of the dominant (right) arm, the expected anticipatory postural motion in the mediolateral plane is expected to be to the right, and the compensatory motion to the left (panel **E**). For the movement (or imagery) of the non-dominant (left) arm, the expected anticipatory postural motion in the mediolateral plane is expected to the left, and the compensatory motion to the right (panel **F**)

The present experiments investigated the effects of unilateral raising of the arm and MI on the postural motions measured just before and after movement onset. Y and O participants stood in canonical stance and raised (or imagined raising) their dominant or non-dominant arm to shoulder level in front of them (Fig. 1). As in Wider et al. (2020), arm movements were either self-initiated or externally triggered. This allowed us to investigate how the ability to control the timing of the focal movement affected the postural activity elicited. The participants’ arm, hip and head motion were recorded using real-time motion tracking. Of interest were the differences in anticipatory and compensatory AP and ML motion accompanying movements of the arm. These differences were examined across two distinct criteria namely the raising of the dominant arm (Experiment 1) or non-dominant arm (Experiment 2). The interaction between age and arm dominance was then analyzed over data from both experiments.

Our expectations of differences in postural motions associated with dominant and non-dominant arm raises were based on the assumption that the dominant arm is favoured, and more practised, in fast or load-bearing movements that generate greater postural perturbation (Bagesteiro and Sainburg 2002; Sainburg and Kalakanis 2000). The movements studied here, as in Wider et al. (2020), were forward arm raises that produced a backward postural perturbation in the anteroposterior plane. In general, anticipatory postural action (moving the body forward) should be more prominent for physical or imagined movements of the dominant arm. Also, unilateral movement of the dominant arm applies a mediolateral postural perturbation to the weaker, non-dominant side. Assuming that this perturbation is more destabilizing than the reverse case, and more so in O than Y (as mediolateral stability is more affected by ageing), anticipatory postural action in the mediolateral direction should also be greater for the dominant arm’s movement or imagery. If O’s postural control is sensitive to this asymmetry, their anticipatory control actions should be more prominent than Y’s in the case of dominant arm movement (or MI).

In their investigation of bilateral arm raises, Wider et al. (2020) studied a self-initiated and an externally triggered task condition. In the latter, the participants knew that a signal to make (or imagine) the movement was imminent but could not predict its exact timing. A key result was that O (but not Y) failed to show anticipatory postural motion in this externally triggered condition. This pointed to an age-related deficit in planning the postural support for actions that must be coordinated with external events. The present experiments retained these task conditions to observe whether postural support patterns change in the case of unilateral action. We expected to see a similar pattern of age-related differences in the case of dominant arm movement (or MI), but as the lateral perturbation due to raising of the non-dominant arm affects the stronger side of the body, this condition may result in a reduced need for mediolateral APM in both self-initiated and externally triggered conditions.

Apart from the unilaterality of arm movements and imagery, the present experiments were identical in protocol to Wider et al’s (2020) study on bilateral arm raises. The key questions addressed across both the studies were (a) does anticipatory postural activity occur during motor imagery, (b) to what extent can anticipatory and compensatory components of postural control fail to co-occur (suggesting separable control processes), and (c) how Y and O differ in postural anticipation when movements (or imagery) are self-initiated or coordinated with environmental triggers. Additionally, the experiments in the present study addressed (d) how Y and O differ in their postural anticipation when the perturbation is induced by physical movement (or planned movement in MI) of the non-dominant or dominant side of the body. The overarching goal across both studies was to inform a model of anticipatory and compensatory postural movement accompanying focal limb motion. This model is presented in the general discussion. Based on this model, we discuss the prospects of using MI-based intervention to strengthen anticipatory postural control in O. Data on both anticipatory and compensatory postural motion are presented for both experiments, but the theoretical focus, especially in the context of MI, is on the anticipatory component.

## Method

### Participants

Twenty-two younger (16 F; age: 19–30 years) and 22 older (12 F, age: 65–89 years) participants were recruited through existing research participation panels consisting of individuals from the university and local communities. The participants did not report any history of balance or neurological disorders and were all right-handed according to the Edinburgh handedness inventory (Oldfield 1971). All had normal or corrected to normal vision. Each participant signed an informed consent form and received a £10 retail voucher for their participation in the session. Ethical approval for the reported research was granted by the Nottingham Trent University College of Business, Law and Social Sciences Research Ethics Committee.

At the start of the session, the participants completed standardized tests of cognitive functioning. The Digit Symbol Substitution test (DSST) from the Wechsler Adult Intelligence Scale-Revised (Wechsler 1981) measured age related differences in speed of processing. The scale has a maximum score of 94, with a higher score indicative of faster processing. The multiple-choice section of the Mill Hill vocabulary scale (MHVS) test (Raven et al. 1988) measured vocabulary (max. score 33). The Y and O groups differed as expected, O significantly outperforming Y on the vocabulary test and Y performing better on the DSST (Salthouse 2010). The participant characteristics are summarized in Table 1.

**Table 1:** Participant characteristic means (with SD in parentheses)

	O	Y
Age (yrs)	70.82 (5.62)	21.86 (3.43)
Height (cm)	170.05 (8.04)	168.14 (10.92)
Weight (kg)	66.18 (13.06)	70.36 (16.20)
EHI	95.45 (9.87)	94.32 (8.39)
Mill Hill	22.86 (3.20)	17.36 (3.97)
DSST	53.77 (10.02)	68.45 (12.62)

*EHI* Edinburgh Handedness Inventory, Mill Hill vocabulary

DSST digit symbol substitution test of information processing speed (from WAIS-R)

Independent *t*-tests showed that Y had significantly smaller DSST scores (t(39.95) = − 4.27, *p* < 0.001) and O had higher vocabulary scores (t(40.17) = 5.06, *p* < 0.0001)

### Apparatus

The participants’ arm and postural motion was recorded using a four-sensor Codamotion tracker (Charnwood Dynamics, Rothley, UK). The system recorded arm motion from active markers placed on the distal end of the middle metacarpal. The system’s pelvic frame was placed horizontally over the posterior superior iliac spine. Markers located on the frame recorded the hip’s motion. The head’s motion was recorded by markers placed on the zygomatic bone. In addition, ground reaction force measurements were also taken, but these are not analyzed here. The experimental trials were delivered by an OpenSesame (Mathôt et al. 2012) script that presented the instructions and the sequence of trials to participants. The script also communicated with Codamotion’s Odin software to start and stop motion data acquisition.

### Procedure

The participants stood barefoot in open stance (heels approximately 10 cm apart) and held a computer mouse in their dominant arm. The instructions for each condition were shown on a monitor placed at eye level 2.5 m in front of the participants.

Each trial started with a recorded voice saying “get ready”. This was followed by a random delay of up to 4000 ms. After this, the voice gave the “go” signal to make or imagine the arm raising movement. The instruction was to raise either the dominant or non-dominant arm until it was horizontal at shoulder level. The participants were asked to click the mouse as they were starting the movement (while their arms were still by their sides) and when they completed the movement (when the arm reached the horizonal position). In the self-initiated movement condition, participants were asked to wait at least 1000 ms after the “go” signal and then start (or imagine starting) arm movement at a time of their own choosing. In the externally triggered condition, the participants were instructed to move (or imagine moving) their arm as soon as they heard the “go” signal.

The MI trials were the same as for the physical movement trials, except that, instead of physically performing the movement, participants closed their eyes and imagined performing the same movement. They clicked the mouse as they started and ended the imagined movement, in the same way as when they performed the physical movements. The instructions emphasized that the participants should imagine what it feels like to make the movement (i.e., the kinaesthetic aspect of imagery). The eyes closed requirement in the MI trials ensured that uncontrolled eye fixation differences could not affect sway in an unpredictable way across task conditions, and that visually focusing on different aspects of the laboratory environment did not distract from the first-person kinesthetic imagery encouraged by the instructions.

All trials were blocked and counterbalanced such that even numbered participants performed in the self-initiated condition first. Conditions that required counterbalancing were mode of execution (physical movement vs MI) and the initiation of movement (self-initiated vs externally triggered), so that there were four blocked conditions in total. Each block consisted of five trials, with one arm movement per trial. The start and end of each trial were controlled by the experimenter. This made it possible for the participants to take frequent breaks. The experimenter took the opportunity to confirm data transfer from the Codamotion server to the data acquisition computer.

In the physical movement conditions, movement recording was preceded by three practice movements. In the MI condition, the participants first made three physical movements and then three imagined movements as practice. This practice was provided to ensure that a physical movement was always completed before imagery regardless of which condition came first. Thus, the participants had a fresh memory of performing the physical movement when engaging in MI (Wider et al. 2020).

### Data analysis

Data analysis considered the anteroposterior (AP) and mediolateral (ML) postural motion of the hip and head segments, and the forward (horizontal) component of arm motion. In the experimental conditions requiring physical movement, arm motion was taken to have started when the arm’s forward velocity exceeded 1 m/s. Postural motion occurring in the preceding 1000 ms period was analyzed as anticipatory postural motion (APM), and motion occurring in the 1000 ms after movement onset was analyzed as compensatory postural motion (CPM). In trials requiring MI rather than physical movement, the participant clicked the mouse button to indicate the start of MI, and the APM and CPM periods were set accordingly. The movement trajectories of all trials were shifted such that the onset of arm motion (or MI) occurred at *t* = 0. Figure 1 shows the conventions used to represent anticipatory and compensatory postural motion in the AP and ML directions.

**Fig. 2:**
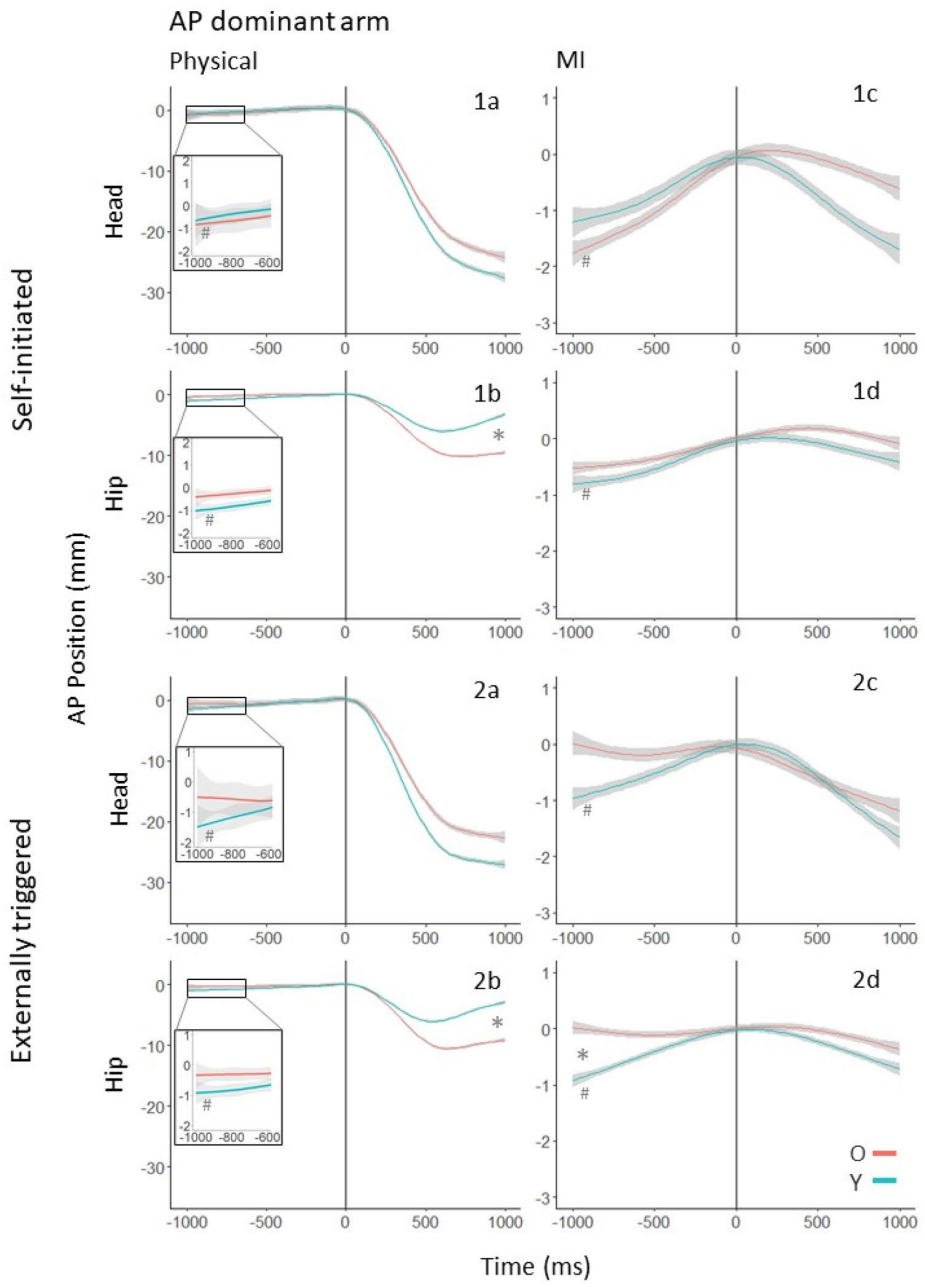
AP postural motion of the dominant (right) arm in the 1000 ms preceding and following the onset of physical (**a**, **b**) and imagined (**c**, **d**) arm raising movements in the self-initiated (1a–d) and externally triggered conditions (2a–d). The position coordinates of all trajectories were shifted such that the onset of arm movement (or MI) occurred at *t* = 0, and the AP position at that moment was set to (0,0). An upward deviation indicates forward movement, and a downward deviation indicates backwards movement. *between Y’s and O’s trajectories indicates a statistically significant difference between the age groups. Trajectories marked with # have slopes significantly different from zero

**Fig. 3:**
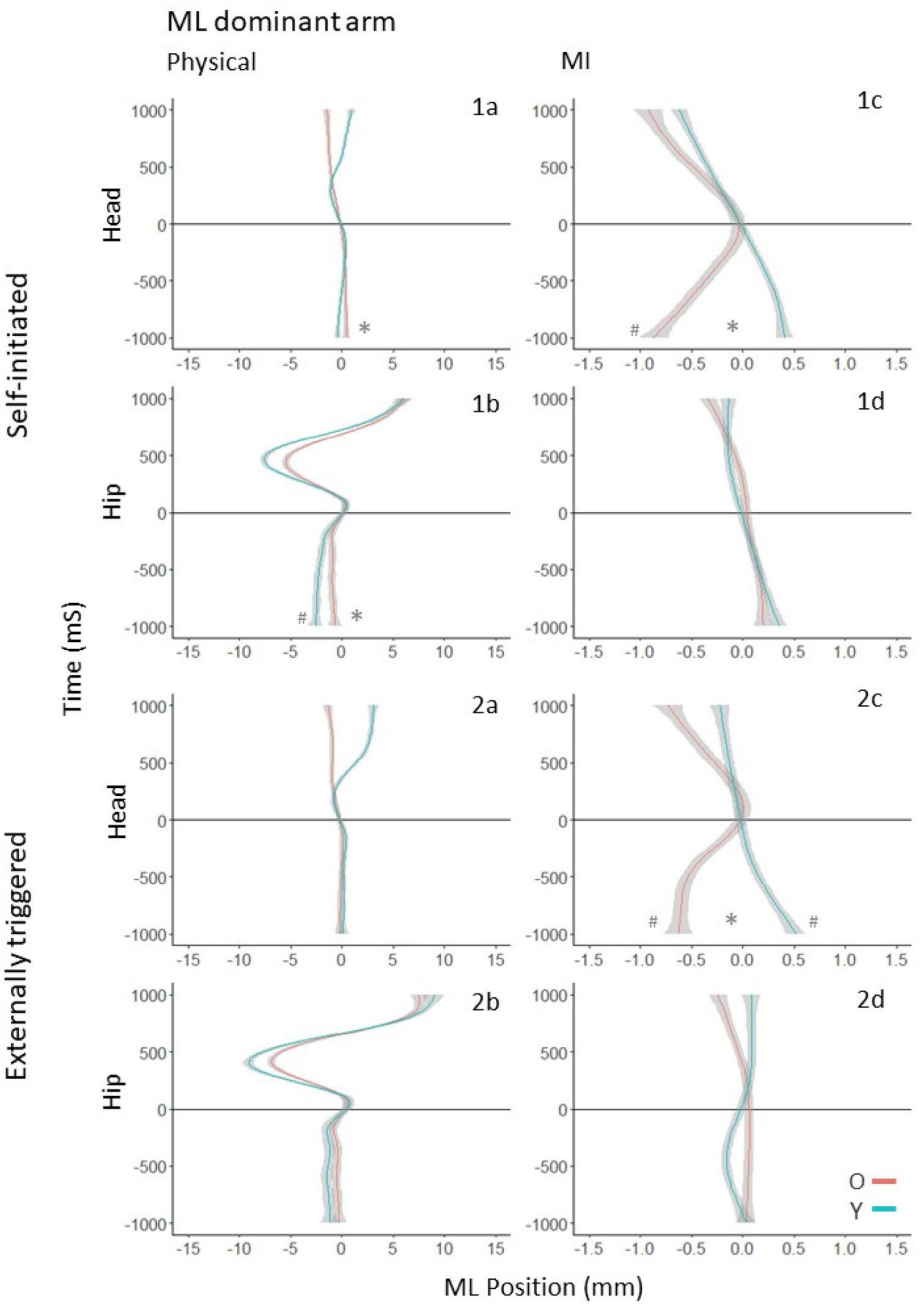
ML postural motion of the dominant (right) arm in the 1000 ms preceding and following the onset of physical (**a**, **b**) and imagined (**c**, **d**) arm raising movements in the self-initiated (1a–d) and externally triggered (2a–d) conditions. The position coordinates of all trajectories were shifted such that the onset of arm movement (or MI) occurred at t = 0, and the ML position at that moment was set to (0,0). Deviations to the right indicate rightward movement, and to the left indicate leftward movement. * between Y’s and O’s trajectories indicates a statistically significant difference between the age groups. Trajectories marked with # have slopes significantly different from zero

**Fig. 4:**
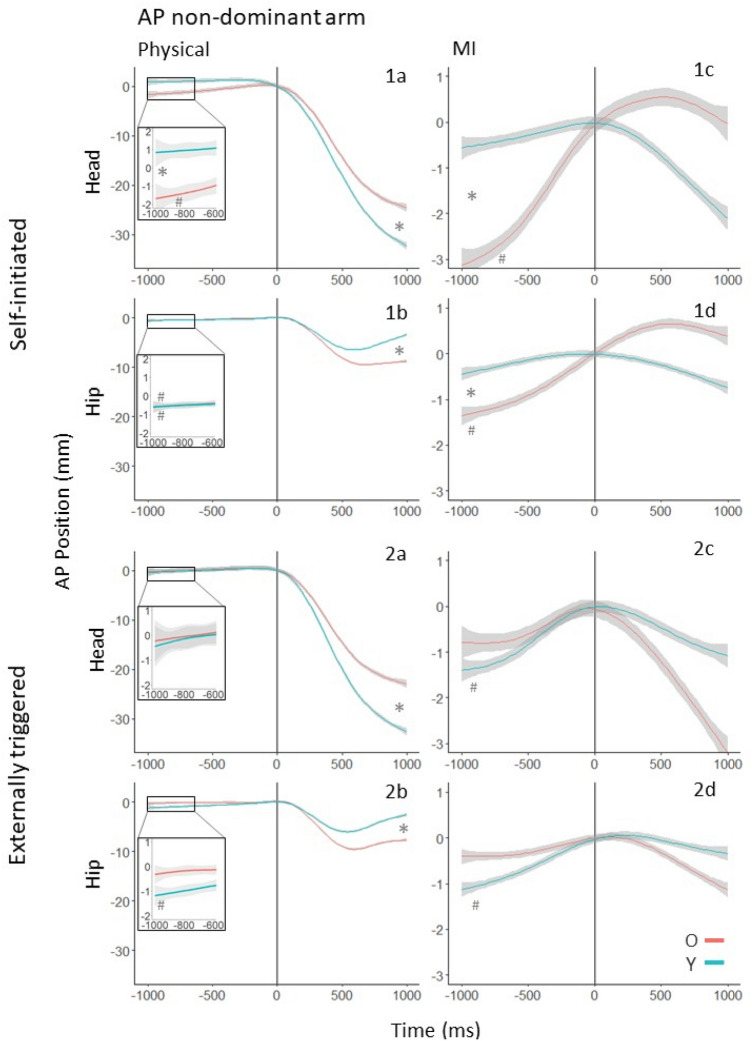
AP postural motion of the non-dominant (left) arm in the 1000 ms preceding and following the onset of physical (**a**, **b**) and imagined (**c**, **d**) arm raising movements in the self-initiated (1a–d) and externally triggered conditions (2a–d). The position coordinates of all trajectories were shifted such that the onset of arm movement (or MI) occurred at *t* = 0, and the AP position at that moment was set to (0,0). An upward deviation indicates forward movement, and a downward deviation indicates backwards movement. * between Y’s and O’s trajectories indicates a statistically significant difference between the age groups. Trajectories marked with # have slopes significantly different from zero

**Fig. 5:**
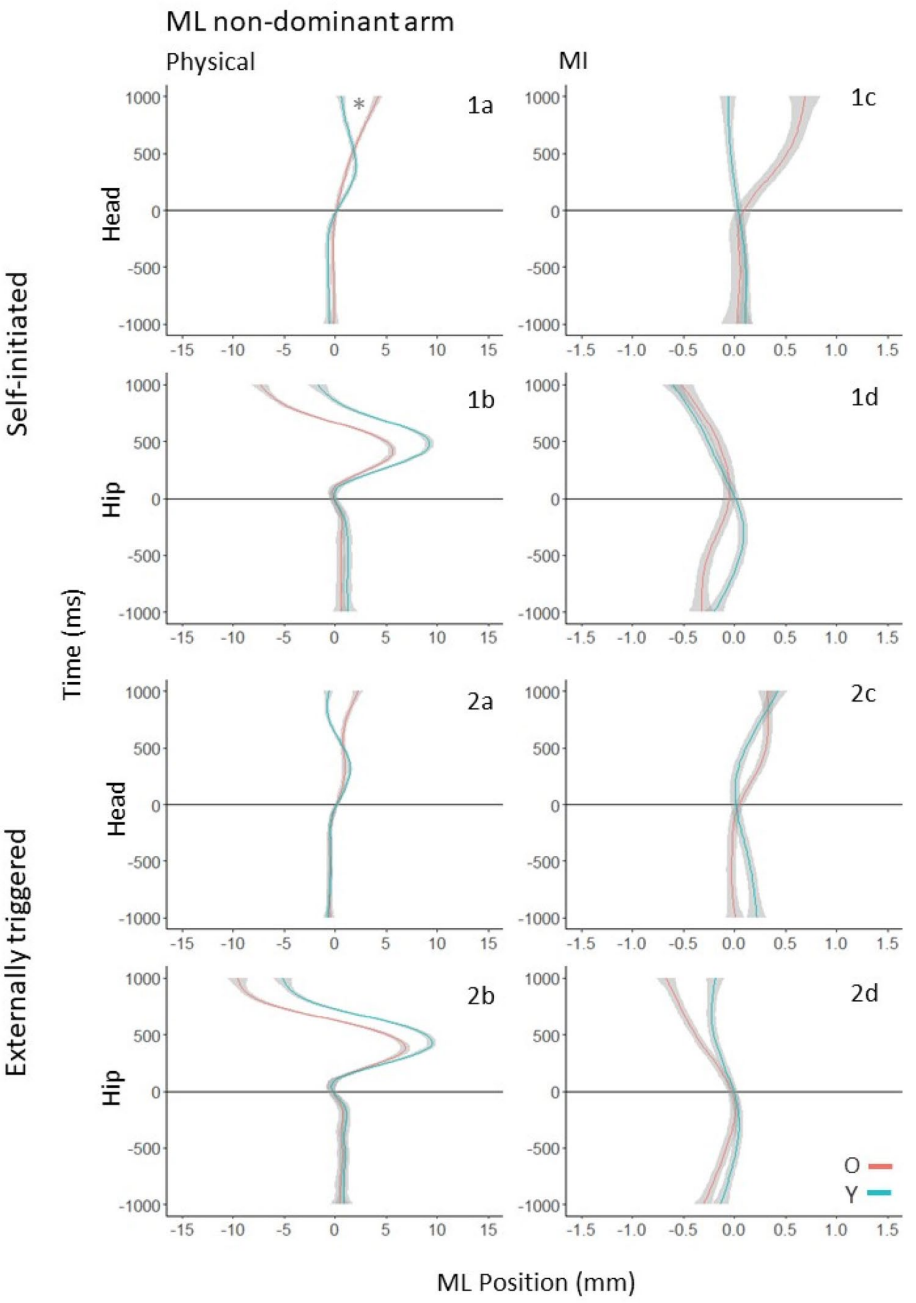
ML postural motion of the non-dominant (left) arm in the 1000 ms preceding and following the onset of physical (**a**, **b**) and imagined (**c**, **d**) arm raising movements in the self-initiated (1a–d) and externally triggered (2a–d) conditions. The position coordinates of all trajectories were shifted such that the onset of arm movement (or MI) occurred at *t* = 0, and the ML position at that moment was set to (0,0). Deviations to the right indicate rightward movement, and to the left indicate leftward movement. * between Y’s and O’s trajectories indicates a statistically significant difference between the age groups. Trajectories marked with # have slopes significantly different from zero

As can be seen in Figs. 2, 3, 4, 5, the APM trajectories in almost all conditions were approximately linear for both head and hip motion (in the cases where trajectories were clearly not linear a 2nd order polynomial was applied to the data). As in Wider et al. (2020), we analyzed APMs using multilevel linear modelling using lme4 (v1.06) in R (Bates et al. 2014; Magezi 2015). First, we analyzed the dominant and non-dominant arm raises separately (reported as Exp. 1 and Exp. 2, respectively). This enabled a detailed understanding of the effects of age and task conditions (self-initiated or externally triggered) for each arm’s movement (or MI). We fit Y and O’s hip or head position data to a varying slope and varying intercept model with time as a fixed effect and participants as a random effect. We referred to this as the test model. In the test model, a positive slope (i.e., time coefficient) in the AP direction indicated forward motion and a positive slope in the ML direction indicated movement to the right. A zero slope before the onset of arm movement indicated the absence of APM. In this case of zero-slope, the data would fit a baseline model that excluded the time coefficient of the test model. Our first hypothesis test compared the test model with the baseline model for Y and O. If the test model fit the data significantly better than the baseline model, we concluded that there was a forward (positive slope) or backward (negative slope) postural motion in the AP direction, and a right (positive slope) or left (negative slope) motion in the ML direction during that time period.

In the next step, we took Y and O’s data together and compared the test model to what we referred to as the theoretical model. The theoretical model added the participants’ age and the interaction between age and time to the test model. If the theoretical model fit the data better (i.e., the time coefficient for Y and O was different), we concluded that Y and O showed different levels of APM. In two instances prior to movement onset, APMs appeared notably curved. In these cases, the same method was applied but with a second-order polynomial fit to curved trajectories. Both non-linear cases were observed under ML motion as follows: the first in Experiment 1, at the hip under externally triggered conditions during MI (Fig. 3, panel 2d), and the second in Experiment 2, at the hip under self-initiated conditions during MI (Fig. 5, panel 1d).

During CPM, head and hip motion had approximately linear trajectories in the majority of MI cases (where trajectories were not linear an order two polynomial was applied to the data) and order two polynomial shape in the case of physical arm movement. In these cases, we simply tested whether trajectories of O and Y were statistically distinguishable. We did not interpret the biomechanics generating the trajectories in terms of the coefficients of our models. Our only interest was whether postural motion of O and Y had different trajectories, and whether head and hip motion phasing differed between O and Y in the time period following movement initiation. As in our analyses of APMs, the theoretical model was a varying intercept and slope model predicting position with age and time (and time^2^ in non-linear cases of arm motion). The interactions between age and each order of time were fixed effects, and participants were a random effect. This theoretical model was compared with a test model that did not include age and its interactions. This comparison tested whether O and Y differed in their postural motion.

The first stage of analysis described so far considered the dominant and non-dominant arm raises separately. In the second stage, we conducted a combined analysis to investigate whether the effects of age and arm dominance interacted. For this, we created a combined model with time, age, arm dominance and the time × age, time × arm dominance, and age × arm dominance interactions as fixed effects and participants as a random effect. We compared this model with another which was identical except that the age x arm dominance interaction was dropped (using the *drop1* function in lme4). We did this separately for self-initiated and externally triggered conditions, physical movements and MI, and the hip and head segments. If this model comparison was significant, we interpreted how the effect of age differed in dominant and non-dominant arm raises. As our theoretical interests were focused on anticipatory control, we have reported the results of this combined analysis only for APM trajectories.

When comparing a pair of linear mixed effects models, we carried out a likelihood ratio test. This calculates the difference in the log of likelihoods of the two models. Under the null hypothesis that the two models are identical, − 2 times the difference in the log of the likelihoods is distributed as a Chi-squared distribution with degrees of freedom equal to the difference in the number of parameters between the two models.

We also analyzed whether O and Y had different arm velocity profiles for physical movements. We did this to eliminate the possibility that any age-related postural differences could have resulted from differences in arm-raising speed between O and Y. For example, slower arm movements by O could reduce or eliminate observable postural motion. We also tested whether O and Y were able to keep their arm still during MI conditions. This checked whether any age-related postural differences in the MI conditions was due to uncontrolled arm movement.

## Results

### Experiment 1: Dominant arm movement and MI

In this experiment, we present the results for the conditions in which movement or MI of the dominant arm occurred. First, we provide a summary of the main results with reference to Figs. 2 and 3. We then present in detail the APM and CPM results for postural motion in the AP direction in the self-initiated and externally triggered conditions. Following this, we present the detailed results for postural motion in the ML direction. Finally, we show that Y and O’s arm velocities did not differ and that both groups were equally able to suppress arm motion during MI.

### Summary of results

We consider postural motion in the AP direction first. For both physical movements and imagery, Y showed APM in both the self-initiated and externally triggered conditions, but O did not in the latter condition (compare Fig. 2, 1a and 2a). For imagery, Y showed APM in both task conditions, but O showed APM only in the self-initiated condition (compare Fig. 2, 1c/d with 2c/d). In the period following the onset of physical arm movement, in both the self-initiated and externally triggered condition, O showed a larger backward CPM (at the hip) than Y (Fig. 2, 1b and 2b). No differences were statistically discernible in the MI conditions.

Next, we consider postural motion in the ML direction. When making physical movements in the self-initiated condition, Y showed APM in the ML direction (Fig. 3, 1b). This was not the case for the head or the hip in the externally triggered condition (Fig. 3, 2a and 2b). O did not show any APM at the head or the hip in self-initiated or externally triggered conditions. When imagining the movements, O showed APM in both conditions but only in the head segment. Y did not show APM in either condition or body segment.

Following the onset of physical or imagined arm movement, no significant ML deviations were detected for either age group in any condition or body segment.

## Detailed results for AP postural motion

### Self-initiated arm movement

#### Anticipatory postural motion

At the hip (Fig. 2, panel 1b), O’s movement was not statistically different to zero (*χ*^2^ (1) = 2.56, *p* = 0.11). Y’s forward motion of 1.08 mm was significantly different to zero (*χ*^2^ (1) = 5.82, *p* = 0.02). However, Y and O’s displacement was not significantly different (*χ*^2^ (2) = 1.59, *p* = 0.46). At the head (Fig. 2, panel 1a), O’s forward motion of 1.28 mm was statistically different to zero (*χ*^2^ (1) = 4.02, *p* = 0.04). Y’s forward motion of 1.04 mm was not significantly different to zero (*χ*^2^ (1) = 1.76, *p* = 0.18). Again, Y and O were not significantly different (*χ*^2^ (2) = 0.18, *p* = 0.91).

Thus, O showed anticipatory forward movement at the head but not the hip, whereas Y showed anticipatory movement at the hip but not the head. However, the two age groups did not differ in APM at either segment.

#### Compensatory postural motion

At the hip (Fig. 2, panel 1b), age, time, time^2^, and the interaction between age and time were significant predictors of AP position. Y and O differed in their motion (*χ*^2^(3) = 19.14, *p* < 0.001). At the head (Fig. 2, panel 1a), time and time^2^ were significant predictors of position, but the two age groups did not differ (*χ*^2^ (3) = 1.73, *p* = 0.63).

O and Y’s compensatory postural motion trajectories accompanying physical arm movement differed at the hip, but not at the head. At the hip, O showed greater backwards displacement than Y. Y moved back towards the pre-movement-onset position following the backwards motion, but O’s hip motion did not show this recovery motion within the 1000-ms window.

### Self-initiated MI

#### Anticipatory postural motion

At the hip (Fig. 2, panel 1d), O’s forward movement of 0.53 mm was not statistically different to zero (*χ*^2^ (1) = 1.90, *p* = 0.17). Y’s forward movement of 0.86 mm was significantly different to zero (*χ*^2^ (1) = 4.59, *p* = 0.03). However, Y and O’s movement did not differ (*χ*^2^ (2) = 0.37, *p* = 0.83). At the head (Fig. 2, panel 1c), O’s forward movement of 1.80 mm was statistically different to zero (*χ*^2^ (1) = 5.72, *p* = 0.02). Y’s forward movement of 1.31 mm was not significantly different to zero (*χ*^2^ (1) = 3.49, *p* = 0.06). Again, the age groups did not differ (*χ*^2^ (2) = 0.37, *p* = 0.83).

As in the physical condition, O showed anticipatory forward movement at the head but not the hip, whereas Y showed anticipatory forward movement at the hip but only slight forward motion at the head. The age groups did not differ from each other in either segment.

#### Compensatory postural motion

At the hip (Fig. 2, panel 1d) and head (Fig. 2, panel 1c), no significant effects were found. O and Y were not distinguishable from each other at either segment.

### Externally triggered arm movement

#### Anticipatory postural motion

At the hip (Fig. 2, panel 2b), O’s forward movement of 0.37 mm was not statistically different to zero (*χ*^2^ (1) = 1.19, p = 0.28). Y’s forward movement of 0.93 mm was significantly different to zero (*χ*^2^ (1) = 6.79, p = 0.01). However, Y and O did not statistically differ in their movement (*χ*^2^ (2) = 1.85, *p* = 0.40). At the head (Fig. 2, panel 2a), O’s forward movement of 0.66 mm was not statistically different to zero (*χ*^2^ (1) = 1.43, *p* = 0.23). Y’s forward movement of 1.76 mm was significantly different to zero (*χ*^2^ (1) = 13.91, *p* < 0.001). Again, movements of Y and O’s were not significantly different (*χ*^2^ (2) = 1.59, *p* = 0.46).

O did not show anticipatory motion at the head or hip, whereas Y did show significant forward motion at both segments. However, O and Y could not be statistically distinguished.

#### Compensatory postural motion

At the hip (Fig. 2, panel 2b), age, time, time^2^, the interaction between age and time, and the interaction between age and time^2^ were significant predictors of AP position. Trajectories of Y and O differed significantly (*χ*^2^ (3) = 19.04, *p* < 0.001). At the head (Fig. 2, panel 2a), age, time, and time^2^ were significant predictors of position. However, Y’s and O’s motion could not be statistically distinguished from each other (*χ*^2^ (3) = 5.23, *p* = 0.16).

O’s and Y’s postural motion trajectories accompanying physical arm movement differed at the hip, O showing less recovery than Y. However, the two age groups were not statistically different.

### Externally triggered MI

#### Anticipatory postural motion

At the hip (Fig. 2, panel 2d.), O’s movement was not statistically different to zero (*χ*^2^ (1) = 0.01, *p* = 0.93). Y’s forward movement of 0.95 mm was significantly different to zero (*χ*^2^ (1) = 10.04, *p* = 0.002). Age affected anticipatory movement, as Y’s and O’s displacement was significantly different (*χ*^2^ (2) = 7.07, *p* = 0.03). At the head (Fig. 2, panel 2c), O’s motion was not statistically different to zero (*χ*^2^ (1) = 0.02, *p* = 0.90). Y’s forward movement of 1.02 mm was significantly different to zero (*χ*^2^ (1) = 5.11, *p* = 0.02). However, Y’s and O’s movements were not statistically distinguishable from each other (*χ*^2^ (2) = 2.17, *p* = 0.34).

O did not show anticipatory motion at the hip or head, while Y did show forward anticipatory motion at both segments; only at the hip were Y and O statistically different.

#### Compensatory postural motion

At the hip (Fig. 2, panel 2d) and head (Fig. 2, panel 2c), no significant effects were found. O and Y were not significantly distinguishable from each other at either segment.

## Detailed results for ML postural motion

### Self-initiated arm movement

### Anticipatory postural motion

At the hip (Fig. 3, panel 1b), O’s movement was not statistically different to zero (*χ*^2^ (1) = 0.15, *p* = 0.70). Y’s rightward movement of 1.93 mm was significantly different to zero (*χ*^2^ (1) = 12.58, *p* < 0.001). Y;s and O’s movements were significantly different (*χ*^2^ (2) = 8.23, *p* = 0.02). At the head (Fig. 3, panel 1a), O’s movement was not statistically different to zero (*χ*^2^ (1) = 0.15, *p* = 0.70). Y’s movement was also not significantly different to zero (*χ*^2^ (1) = 2.77, *p* = 0.10). However, Y’s and O’s movements were significantly different (*χ*^2^ (2) = 6.38, *p* = 0.04).

At the hip, O showed no APM, whereas Y showed APM to the right. This age difference was significant. At the head, O showed a slight leftward tendency, and Y a rightward tendency. Neither of these trajectories was significantly different to zero, but they were significantly different from each other.

#### Compensatory postural motion

At the hip (Fig. 3, panel 1b) and head (Fig. 3, panel 1a), no significant effects were found. O and Y were not significantly distinguishable from each other at either segment.

### Self-initiated MI

#### Anticipatory postural motion

At the hip (Fig. 3, panel 1d), no significant results were found. At the head (Fig. 3, panel 1c), O’s rightward movement of 0.92 mm was statistically different to zero (*χ*^2^ (1) = 6.78, *p* = 0.01). Y’s movement was not statistically different to zero (*χ*^2^ (1) = 2.32, *p* = 0.13). Age was found to have an effect, as Ys and O’s displacements significantly differed (*χ*^2^ (2) = 8.96, *p* = 0.01).

O, but not Y, exhibited anticipatory rightward motion at the head. This difference between O and Y was significant. At the hip, both age groups showed no difference in APM.

#### Compensatory postural motion

At the hip (Fig. 3, panel 1d) and head (Fig. 3, panel 1c), no significant effects were found. O and Y were not significantly distinguishable from each other at either segment.

### Externally triggered arm movement

#### Anticipatory postural motion

At the hip (Fig. 3, panel 2b) and head (Fig. 3, panel 2a), no significant effects were found. O and Y were not significantly distinguishable from each other or zero at either segment.

#### Compensatory postural motion

At the hip (Fig. 3, panel 2b) and head (Fig. 3, panel 2a), no significant effects were found. O and Y were not significantly distinguishable from each other at either segment.

### Externally triggered MI

#### Anticipatory postural motion

At the hip (Fig. 3, panel 2d), no significant effects were found. At the head (Fig. 3, panel 2c), O’s right trajectory of 0.63 mm was statistically different to zero (*χ*^2^ (1) = 3.88, *p* = 0.049). Y’s left movement of − 0.58 mm was also significantly different to zero (*χ*^2^ (1) = 4.12, *p* = 0.04). Age was found to have an effect as Y and O’s displacement was significantly different (*χ*^2^ (2) = 7.97, *p* = 0.02).

Both O and Y exhibited significant anticipatory motion: O moving to the right and Y the left. The difference between O and Y was significant.

#### Compensatory postural motion

At the hip (Fig. 3, panel 2d) and head (Fig. 3, panel 2c), no significant effects were found. O and Y were not significantly distinguishable from each other at either segment.

##### Arm movement peak velocity and its latency

In those conditions in which the arm was physically raised, we tested for any age-related differences in the arm’s peak velocity and its timing.

The theoretical model was a varying intercept and slope model predicting the arm’s peak AP velocity and latency. Age and time were fixed effects and participants a random effect. We compared this model with a test model that lacked the age coefficient.

In the SI condition, there was no difference between Y and O’s peak velocity (*χ*^2^ (1) = 0.82, *p* = 0.36) or its latency (*χ*^2^ (1) = 3.38, *p* = 0.07). In the externally triggered condition, there was no difference between Y and O’s peak velocity (*χ*^2^ (1) = 0, *p* = 0.998) or its latency (*χ*^2^ (1) = 3.65, *p* = 0.06).

##### Arm motion during MI

In the case of the MI conditions, we tested whether the arm had systematic forward or backward motion in the 1000 ms before or after the start of MI (as indicated by participants’ mouse click). The test model was a varying intercept and slope model predicting the arm’s AP position with time as a fixed effect and participants as a random effect. We compared this model with a baseline model that removed the time coefficient. We rejected the null hypothesis (that there was no AP displacement) if the test model ft the data significantly better than the null model.

In the self-initiated condition, O showed no significant arm motion in the pre-MI period (*χ*^2^ (1) = 1.34, *p* = 0.25) and Y showed marginally significant forward motion (*χ*^2^ (1) = 3.76, *p* = 0.053). During the MI period, O showed no significant arm motion (*χ*^2^ (1) = 0.01, *p* = 0.90), and neither did Y (*χ*^2^ (1) = 0.36, *p* = 0.55).

In the externally triggered condition, O showed no significant arm motion in the pre-MI period (*χ*^2^ (1) = 1.14, *p* = 0.29). Y did show significant forward motion (*χ*^2^ (1) = 6.89, *p* = 0.01), however, the magnitude of 1.20 mm was comparable to the 1.02 mm of head motion recorded in this time period. During the MI period, O showed no significant arm motion (*χ*^2^ (1) = 3.19, *p* = 0.07), and neither did Y (*χ*^2^ (1) = 1.83, *p* = 0.18).

These results demonstrate that arm motion and postural motion (recorded from the upper body) were comparable. We concluded from this that O and Y were equally able to inhibit focal arm movement MI onset.

### Experiment 2: Non-dominant arm movement and MI

In this experiment, we present the results for the conditions in which movement or MI of the non-dominant arm occurred. As for Experiment 1, we first present the APM and CPM results for postural motion in the AP direction in the self-initiated and externally triggered conditions. Following this, we present the results for postural motion in the ML direction. Finally, we provide a summary of all the results with reference to Figs. 4 and 5.

The experimental protocol and set up, as well as the methodological collection and analysis of data, were the same between Experiment 1 and 2, with the exception that in Experiment 2 participants raised their non-dominant arm. First, we provide a summary of the main results with reference to Figs. 4 and 5. Next, we present detailed results for APM and CPM in the AP and ML directions. Finally, we show that arm velocity and suppression of arm motion during MI were not different between Y and O.

#### Summary of results

We consider postural motion in the AP direction first. When making physical movements in the self-initiated condition, O showed APM at both segments, but Y did so only at the hip (Fig. 4, 1a and 1b). In the externally triggered condition, O did not show APM at either segment, but Y did at the hip (Fig. 4, 2a and 2b). When imagining the movements, O but not Y showed significant APM in the self-initiated condition (Fig. 4, 1c and 1d). In the externally triggered case, Y but not O showed significant APM at both segments (Fig. 4, 2c and 2d).

Following the onset of physical arm movement, in both the self-initiated and externally triggered conditions, O showed greater backward movement at the hip and less at the head than Y (Fig. 4, 1a and 1b). In the MI conditions, O’s and Y’s trajectories did not have statistically significant deviations or mutual differences.

For postural motion in the ML direction, there was no evidence of APM in either condition or body segment in Y or O. The results were the same in the case of CPM after arm movement or MI onset. As shown in Fig. 5, 1a, there was a significant difference between Y and O at the head in the case of physical movement under self-initiated conditions. As neither trajectory deviated significantly from zero, we did not interpret this difference.

## Detailed results for AP postural motion

### Self-initiated arm movement

#### Anticipatory postural motion

At the hip (Fig. 4, panel 1b), O’s forward movement of 0.60 mm was statistically different to zero (*χ*^2^ (1) = 4.30, *p* = 0.04). Y’s forward movement of 0.51 mm was also significantly different to zero (*χ*^2^ (1) = 4.54, *p* = 0.03). However, Y’s and O’s movements were not significantly different from each other (*χ*^2^ (2) = 0.47, *p* = 0.79). At the head (Fig. 4, panel 1a), O’s forward movement of 2.19 mm was statistically different to zero (*χ*^2^ (1) = 4.77, *p* = 0.03). Y’s movement was not significantly different to zero (*χ*^2^ (1) = 0.01, *p* = 0.93). Y’s and O’s trajectories were significantly different (*χ*^2^ (2) = 14.74, *p* < 0.001).

O and Y exhibited similar anticipatory forward motion at the hip, and the age difference was not significant. At the head, O showed forward motion while Y did not show any deviation from zero. This age difference was also significant.

#### Compensatory postural motion

At the hip (Fig. 4, panel 1b), age, time, time^2^, and the interaction between age and time were significant predictors of AP position. Y’s and O’s trajectories were statistically distinguishable (*χ*^2^ (3) = 16.10, *p* = 0.001). At the head (Fig. 4, panel 1a), age, time, time^2^, and the interaction between age and time were significant predictors. Again, Y’s and O’s trajectories were statistically distinguishable (*χ*^2^ (3) = 15.94, *p* = 0.001).

O’s and Y’s trajectories differed at both the head and hip segments. At the hip, Y showed backwards motion before moving back towards the pre-movement-onset position. O showed more backwards motion than Y, but no tendency return to baseline within the observation window. At the head, O and Y showed similar backwards motion; however, Y showed greater backwards displacement than O.

### Self-initiated MI

#### Anticipatory postural motion

At the hip (Fig. 4, panel 1d), O’s forward trajectory of 1.35 mm was statistically different to zero (*χ*^2^ (1) = 7.17, *p* = 0.01), but Y’s movement was not significantly different to zero (*χ*^2^ (1) = 1.66, *p* = 0.20). Y’s and O’s displacements were significantly different (*χ*^2^ (2) = 6.31, *p* = 0.04). At the head (Fig. 4, panel 1c), O’s forward trajectory of 3.35 mm was statistically different to zero (*χ*^2^ (1) = 11.62, *p* < 0.001), but Y’s movement was not significantly different to zero (*χ*^2^ (1) = 1.00, *p* = 0.32). Again, Y’s and O’s displacements were significantly different from each other (*χ*^2^ (2) = 6.81, *p* = 0.03).

O and Y were statistically distinguishable in their movement trajectories at the hip and head. At both segments, O showed significant forward motion, whereas Y did not.

#### Compensatory postural motion

At the hip (Fig. 4, panel 1d) and head (Fig. 4, panel 1c), no significant effects were found. O and Y were not significantly distinguishable from each other at either segment.

### Externally triggered arm movement

#### Anticipatory postural motion

At the hip (Fig. 4, panel 2b), O’s movement was not statistically different to zero (*χ*^2^ (1) = 0.85, *p* = 0.36). Y’s forward movement of 1.17 mm was significantly different to zero (*χ*^2^ (1) = 9.24, *p* = 0.002). However, Y’s and O’s displacements were not significantly different (*χ*^2^ (2) = 4.76, *p* = 0.09). At the head (Fig. 4, panel 2a), no significant effects were found.

O did not show significant motion at the hip or head. Y did show significant forward motion at the hip only, but O and Y did not differ significantly.

#### Compensatory postural motion

At the hip (Fig. 4, panel 2b), age, time, time^2^, the interaction between age and time, and the interaction between age and time^2^ were significant predictors of position. Y’s and O’s trajectories differed significantly (*χ*^2^ (3) = 13.47, *p* = 0.004). At the head (Fig. 4, panel 2a), age, time, time^2^, the interaction between age and time, and the interaction between age and time^2^ were significant predictors of position. Again, Y’s and O’s trajectories differed significantly (*χ*^2^ (3) = 8.31, *p* = 0.04).

At the hip, O and Y show backwards motion prior to forward motion bringing the body back to baseline. The head shows similar trajectories for O and Y but Y’s displacement was greater.

### Externally triggered MI

#### Anticipatory postural motion

At the hip (Fig. 4, panel 2d), O’s movement was not statistically different to zero (*χ*^2^ (1) = 0.74, *p* = 0.39). Y’s forward movement of 1.17 mm was significantly different to zero (*χ*^2^ (1) = 7.70, *p* = 0.01). However, Y’s and O’s displacements were not significantly different from each other (*χ*^2^ (2) = 1.46, *p* = 0.48). At the head (Fig. 4, panel 2c), O’s movement was not statistically different to zero (*χ*^2^ (1) = 1.14, *p* = 0.29). Y’s forward movement of 1.66 mm was significantly different to zero (*χ*^2^ (1) = 6.00, *p* = 0.01). However, again, Y’s and O’s displacements were not significantly different (*χ*^2^ (2) = 0.45, *p* = 0.80).

O showed no significant motion at the hip or head, whereas Y did. However, in neither case were O and Y statistically distinguishable.

#### Compensatory postural motion

At the hip (Fig. 4, panel 2d) and head (Fig. 4, panel 2c), no significant effects were found. O and Y were not significantly distinguishable from each other at either segment.

## Detailed results for ML postural motion

### Self-initiated arm movement

#### Anticipatory postural motion

At the hip (Fig. 5, panel 1b) and head (Fig. 5, panel 1a), no significant effects were found. O and Y were not significantly distinguishable from each other or zero at either segment.

#### Compensatory postural motion

At the hip (Fig. 5, panel 1b), no significant effects were found. At the head (Fig. 5, panel 1a), Y’s and O’s trajectories were statistically distinguishable (*χ*^2^ (3) = 14.29, *p* = 0.003).

Y and O show rightwards motion immediately after the onset of the movement, with Y showing a steeper movement trajectory than O before returning back to baseline. O continue on a steady rightwards trajectory and did not bring themselves back to baseline within the 1000 ms time window.

### Self-initiated MI

#### Anticipatory postural motion

At the hip (Fig. 5, panel 1d) and head (Fig. 5, panel 1c), no significant effects were found. O and Y were not significantly distinguishable from each other or zero at either segment.

#### Compensatory postural motion

At the hip (Fig. 5, panel 1d) and head (Fig. 5, panel 1c), no significant effects were found. O and Y were not significantly distinguishable from each other at either segment.

### Externally triggered arm movement

#### Anticipatory postural motion

At the hip (Fig. 5, panel 2b) and head (Fig. 5, panel 2a), no significant effects were found. O and Y were not significantly distinguishable from each other or zero at either segment.

#### Compensatory postural motion

At the hip (Fig. 5, panel 2b) and head (Fig. 5, panel 2a), no significant effects were found. O and Y were not significantly distinguishable from each other at either segment.

### Externally triggered MI

#### Anticipatory postural motion

At the hip (Fig. 5, panel 2d) and head (Fig. 5, panel 2c), no significant effects were found. O and Y were not significantly distinguishable from each other or zero at either segment.

#### Compensatory postural motion

At the hip (Fig. 5, panel 2d) and head (Fig. 5, panel 2c), no significant effects were found. O and Y were not significantly distinguishable from each other at either segment.

##### Arm movement peak velocity and its latency

In the self-initiated condition, there was no difference between Y and O’s peak velocity (*χ*^2^ (1) = 0.53, *p* = 0.47) or its latency (*χ*^2^ (1) = 0.34, *p* = 0.56). In the externally triggered condition, there was no difference between Y and O’s peak velocity (*χ*^2^ (1) = 0.16, *p* = 0.69), or its latency (*χ*^2^ (1) = 1.01, *p* = 0.31).

##### Arm motion during MI

In the self-initiated condition, O did show significant arm motion in the pre-MI period (*χ*^2^ (1) = 8.14, *p* = 0.004); however, the magnitude of 2.38 mm was comparable to the 3.35 mm of head motion recorded in this period. Y showed no significant arm motion (*χ*^2^ (1) = 2.98, *p* = 0.08). During the MI period, O showed no significant arm motion (*χ*^2^ (1) = 2.89, *p* = 0.09) and neither did Y (*χ*^2^ (1) = 1.44, *p* = 0.22).

In the externally triggered condition, O showed no significant arm motion in the pre-MI period (*χ*^2^ (1) = 2.69, *p* = 0.10). Y did show significant arm motion (*χ*^2^ (1) = 5.48, *p* = 0.02); however, the magnitude of 1.26 mm was comparable to the 1.66 mm of head sway recorded during this period. During the MI period, O showed no significant arm motion (*χ*^2^ (1) = 2.71, *p* = 0.10) and neither did Y (*χ*^2^ (1) = 0.48, *p* = 0.49).

These results suggests that arm motion and postural motion (recorded from the upper body) were comparable and that both O and Y were about equally able to inhibit arm movement before and after MI onset.

## Cross-experiment analysis of changes in age effects for dominant and non-dominant arm raises

Data from Experiments 1 and 2 were combined and a full model including time, age, arm dominance, and all three two-way interactions was compared with a model that excluded the age × arm dominance interaction. Where this model comparison was significant, we interpreted how the effect of age differed for the two arms. Only the results for anticipatory postural motion are presented. Detailed results follow a summary of findings.

### Summary of results

The difference in postural anticipation between Y and O did not have the same pattern for the dominant and non-dominant arms. We consider physical movements first. For self-initiated movements, the key difference in the AP direction was inter-segmental. O showed a greater head APM whereas Y showed a greater hip APM. For externally triggered movements, O did not show any APM for either arm. Y did show APM, at both head and hip for the dominant arm and at the hip for the non-dominant arm. In the ML direction, Y but not O showed APM for the dominant arm in the self-initiated condition. In the self-initiated condition, O did not show APM but Y showed APM at the hip segment. Thus, the results for physical movement suggest that O tended to use a hip strategy whereas Y deployed an ankle strategy. O’s lack of APM in the externally triggered condition spanned all conditions.

Next, we consider MI. In the self-initiated condition, O showed larger APM in the AP direction for the non-dominant arm. In the externally triggered condition, O did not show APM in any condition whereas Y did for both arms. In the ML direction, O showed large APM at the head in both task conditions in the case of the dominant arm, and neither age groups showed prominent APM in the other cases. The results for MI suggest that O produced more prominent APM than Y for the dominant arm, and there was again evidence of O’s use of a hip strategy.

## Detailed results for AP postural motion

### Self-initiated arm movement

*Hip*. The model comparison was significant (*χ*^2^ (1) = 174.93, *p* < 0.001). Y showed a larger APM than O for the dominant arm raise. This age difference was not prominent for the non-dominant arm. Compare Fig. 2, 1b and Fig. 4, 1b.

*Head*. The model comparison was significant (*χ*^2^ (1) = 709.55, *p* < 0.001). For the dominant arm, there was little difference in APM between Y and O. For the non-dominant arm, O made a larger forward APM than Y. Compare Fig. 2, 1a and Fig. 4, 1a.

### Self-initiated MI

*Hip and Head*. The model comparisons were significant (*χ*^2^ (1) = 573.14, *p* < 0.001; *χ*^2^ (1) = 539.17, *p* < 0.001). For the dominant arm, there was little difference in APM between Y and O. For the non-dominant arm, there was an age-related difference–O made a larger forward APM at both segments than Y. Compare Fig. 2, 1c, 1d and Fig. 4, 1c, 1d.

### Externally triggered arm movement

*Hip.* The model comparison was significant (*χ*^2^ (1) = 34.89, *p* < 0.001). Y showed a larger forward APM than O for both arms, but the difference was larger for the non-dominant arm. Compare Fig. 2, 2b and Fig. 4, 2b.

*Head*. The model comparison was significant (*χ*^2^ (1) = 15.17, *p* < 0.001). Y and O only differed for the dominant arm, where Y showed a slightly larger forward APM. Compare Fig. 2, 2a and Fig. 4, 2a.

### Externally triggered MI

*Hip.* The model comparison was not significant (*χ*^2^ (1) = 0.08, *p* = 0.77). The difference between Y and O did not change between the dominant and non-dominant arms. O did not show APM whereas Y did. Compare Fig. 2, 2d and Fig. 4, 2d.

*Head.* The model comparison was significant (*χ*^2^ (1) = 5.54, *p* = 0.02). O did not show APM whereas Y did. The difference was more prominent for the dominant arm. Compare Fig. 2, 2c and Fig. 4, 2c.

## Detailed results for ML postural motion

### Self-initiated arm movement

*Hip and Head*. The model comparisons were significant (*χ*^2^ (1) = 1079.2, *p* < 0.001; *χ*^2^ (1) = 0.52, *p* < 0.01). Y but not O made a rightward APM for the dominant arm. There was no APM for the non-dominant arm. Compare Fig. 3, 1a, 1b and Fig. 5, 1a, 1b.

### Self-initiated MI

*Hip*. The model comparison was significant (*χ*^2^ (1) = 100.30, *p* < 0.001). For the dominant arm, there was little difference in APM between O and Y. For the nondominant arm, O showed greater rightward movement than Y. Compare Fig. 3, 1d and Fig. 5, 1d.

*Head*. The model comparison was significant (*χ*^2^ (1) = 602.99, *p* < 0.001). Y did not show APM for either arm, but O showed a rightward APM for the dominant arm. Compare Fig. 3, 1c and Fig. 5, 1c.

### Externally triggered arm movement

*Hip*. The model comparison was significant (*χ*^2^ (1) = 731.12, *p* < 0.001). Y showed a slight rightward APM for the dominant arm, but O did not show APM for either arm. Compare Fig. 3, 2b and Fig. 5, 2b.

*Head*. The model comparison was significant (*χ*^2^ (1) = 60.71, *p* < 0.001), but there was no clear difference between Y and O. Compare Fig. 3, 2a and Fig. 5, 2a.

### Externally triggered MI

*Hip*. The model comparison was significant (*χ*^2^ (1) = 112.64, *p* < 0.001), but Y and O did not show clear differences in APM. Compare Fig. 3, 2d and Fig. 5, 2d.

*Head*. The model comparison was significant (*χ*^2^ (1) = 406.15, *p* < 0.001). O showed a clear rightward APM for the dominant arm but not for the non-dominant arm. Y did not show clear APM for either ARM. Compare Fig. 3, 2c and Fig. 5, 2c.

## General discussion

When an arm is raised in front of the body, the CG moves forward, necessitating a backward CPM to maintain stability as the movement occurs (Bouisset and Zattara 1981, 1987b, 1988, 1990; Friedli et al. 1988; Mouchnino et al.^1990^; Ramos and Stark 1990; Rogers and Pai 1990). For forward movements of either arm, CPMs of both Y and O showed backward postural movement in the first 500 ms following movement onset in both conditions and experiments (Figs. 2, 4). Following this, Y’s but not O’s hip motion reversed direction, as head motion continued backwards in both groups. This pattern is consistent with the use of backward bending of the trunk to regulate CG (Martin 1967) in Y, as was also observed by Wider et al. (2020) for bilateral arm raises. In the ML direction, CPMs to the left for dominant (right) arm raises (Fig. 3), and to the right for non-dominant (left) arm raises (Fig. 5) were observed at the hip in both task conditions. This suggests that unilateral forward arm raises generate a lateral perturbation that is counteracted by a hip movement to the inactive side.

Any anteroposterior APM that occurs prior to forward arm movements is expected to be in the forward direction, opposite to the backward CPMs observed during the movements themselves (Bleuse et al. 2006; Cordo and Nashner 1982). Y showed a consistent pattern of forward APM, particularly at the hip segment, across task and arm dominance conditions. In contrast, O did not show forward APM in the externally triggered condition for either arm. In the self-initiated condition, O’s APM occurred at the head segment, suggesting a difference from Y in relying more on a hip strategy. The absence of O’s APM in the externally triggered condition replicated and extended the pattern seen by Wider et al. (2020) in the case of bilateral arm raises. In the externally triggered condition, a random delay between the ready and go signals did not allow participants to predict the exact time of movement onset. O’s consistent lack of APM under these conditions suggests a lack of preparatory postural action when an expected movement must be coordinated with an external perceptual event. As discussed shortly, the results of the MI conditions corroborate this.

Any mediolateral APM preceding dominant (right) arm movement would be to the right, and it would be to the left in the case of non-dominant (left) arm movement. In the case of right arm movement (Exp. 1), the only indication of APM to the right was seen in Y at the hip in the self-initiated condition (Fig. 3, 1b). Y did not show lateral APM in the externally triggered condition, and O did not in externally triggered or self-initiated conditions. In the case of left arm movement (Exp. 2), neither group showed any lateral APM. We return to these results in the context of the MI results discussed next.

We turn next to the postural motions observed when the arm movements were imagined rather than executed. As seen in Wider et al. (2020), O did not show anteroposterior APM when MI of either arm occurred in the externally triggered condition. In the self-initiated condition, O’s APM was again predominantly at the head segment. This trend was also seen for APM in the mediolateral direction, where only O showed APM and only at the head segment. This consistent pattern suggests a hip strategy in O’s anticipatory control. O’s consistent absence of APM in the externally triggered condition suggests a lack of postural preparation when the planned movement’s onset must be coordinated with an external event. It is likely linked to the sensory integration deficits that characterise postural control in older age (Teasdale et al. 1991; Redfern et al. 2001). Everyday life includes numerous instances in public places or social settings in which a particular movement can be foreseen and planned for, but its execution must await the arrival of an external sensory signal. For example, observing an acquaintance approaching can prime the motor planning of reaching for a handshake. However, the start of the movement must await a comfortable inter-personal distance and facial or linguistic signals. O have been shown previously to produce smaller and more delayed APA (Inglin and Woollacott 1988; Woollacott and Manchester 1993). The observed absence of anteroposterior APM during MI suggests that there is a general age-related deficit in the planning of postural support in the externally triggered condition, irrespective of which arm (or both) is deployed.

We did not observe any indications of mediolateral APM by Y in any of the MI conditions in either experiment. Y’s mediolateral APM preceding physical movement of the dominant arm (Exp. 1) in the self-initiated condition (Fig. 3, 1b) suggests that mediolateral APM is indeed a feature of postural support for unilateral arm raises to the front of the body. This component may be small enough that it was not expressed by Y during MI trials. O, however, did show mediolateral APM for MI of the dominant arm in both the self-initiated and externally triggered conditions of Exp. 1 (Fig. 3, panels 1c and 2c). This suggests that O did plan postural support for a mediolateral perturbation when imagining forward movements of the dominant arm. O, like Y, did not show mediolateral APM when the non-dominant arm was raised in Exp. 2. One reason for this may be that O needed to plan a mediolateral APM when imagining raising the dominant arm because the expected perturbation would be to the weaker, non-dominant side. It is worth noting in this context that the ML direction is considered more important than AP for stepping out in case of falling (Rogers and Mille 2003; Lord et al. 1999). The anticipatory head (but not hip) motion to the dominant side that was observed in O in Exp. 1 is consistent with the use of a hip strategy (Horak and Nashner 1986) to reduce the perceived likelihood of needing to step to the left. When the MI was of the non-dominant arm, the mediolateral perturbation to the stronger, dominant side may have been absorbed without the need for APM. O are known to be more prone to using a hip strategy (Inglin and Woolacott 1988; Lin et al. 2004; Bleuse et al. 2006), but, in fact, this may be more destabilizing in the context of MI than APM involving a shift of hip position.

It should be noted that the MI trials were conducted under eyes closed conditions. In view of O’s generally greater reliance on visual feedback, this could have affected Y and O differently. However, the key age effects were consistent across physical and MI conditions. For example, O’s lack of anteroposterior APM under externally triggered conditions occurred in both physical and MI conditions and for both the dominant and non-dominant arm. This was also the case in Wider et al. (2020) for bilateral arm raises. O’s mediolateral APM in MI trials occurred only in the case of the dominant arm and was consistent with O’s greater vulnerability to a perturbation to the weaker side of the body. Thus, differences in visual information did not materially impact the key results.

Further work is needed to closely inspect whether O’s APMs have a greater tendency to incorporate leaning of the upper body consistent with a hip strategy. The overall pattern of Y and O’s postural motion was similar for the unilateral movements of dominant and non-dominant arms when compared to the bilateral movements studied in Wider et al. (2020). However, O showed stronger anteroposterior APM, involving both head and hip, preceding MI of the non-dominant arm compared to the dominant arm (compare Figs. 4 and 2, panels 1c and 1d). Also, as noted above, O but not Y showed mediolateral APM preceding MI of the dominant arm. In both these conditions, the expected postural perturbation impacted the weaker, non-dominant side of the body. O appear to have been sensitive to this in their postural planning.

It is important to note that, as in Wider et al. (2020), we have been careful to distinguish EPAs, APAs and CPAs, usually studied as patterns of muscle activation, from the APMs and CPMs of our kinematic analysis. The presence of postural motion may be an indication of muscle activation generating it, or the absence of muscle activity to counteract motion due to gravity. Also, there could be periods of opposing muscle activity within the anticipatory period (potentially indicating opposing EPA and APA) even as the resulting motion is approximately linear until the onset of limb movement or MI. Similarly, the absence of postural motion may signal the absence of muscular effort, or it may be the result of muscle activity that did not produce motion (e.g., co-contraction of agonist–antagonist groups). We focused on postural motion as it is the change in body position that affects stability, regardless of the pattern of muscle activity that serves to generate it. Future work needs to map the correspondence between the observed anticipatory motion and the pattern of postural muscle activity. A surface EMG approach to this is feasible for the physical movement conditions, but may be challenging if activity is attenuated during MI.

## Implications

The present results reinforce Wider et al.’s (2020) suggestion that the anticipatory and compensatory components of postural control are separable, whereas the focal movement and compensatory postural adjustment are closely coordinated. Anticipatory postural actions do not always occur, and in the case of O, they are less likely to occur when the planned movement must coordinate with external events. Also, they are comparatively stronger when the expected postural perturbation impacts the weaker, non-dominant side of the body. The occurrence of APMs during MI, and their modulation based on task conditions suggests MI could be an effective means of providing training in anticipatory postural control. Recent research is showing that MI training may benefit a number of measures of postural stability in O (Nicholson et al. 2019; Oh and Choi 2021) and neurological patients (Cho et al. 2013). So far, there has not been a specific focus on anticipatory postural control tasks and tests. Developing such focus in rehabilitation studies using MI may augment O’s postural support of limb movements and potentially mitigate the loss of coordination between anticipatory postural control and environmental events.

**Fig. 6:**
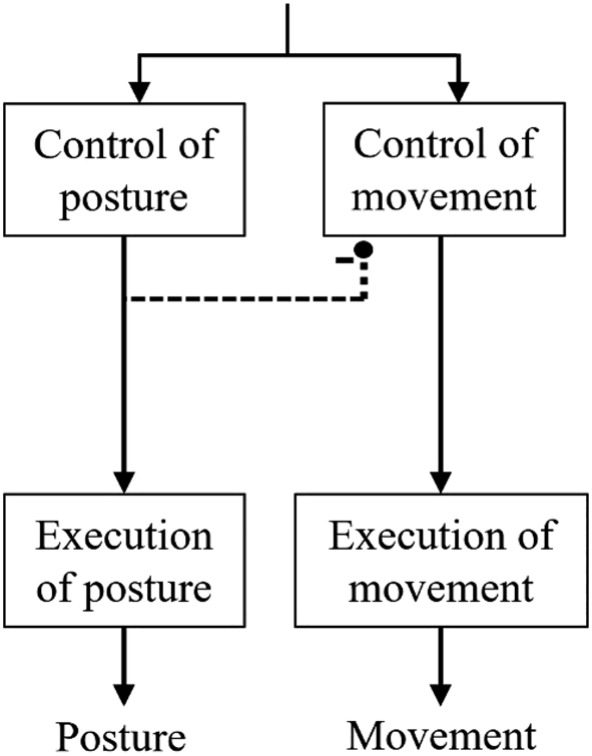
Parallel descending pathways of central origin for the control of focal movement and the postural support for the movement

One reason why a specific focus on anticipatory postural control has not developed is that theoretical synthesis of the role of postural control in the overall architecture of planning physical or imagined limb movements has not been prioritized since Massion (1992) summarized the control of focal movement execution and its postural support as parallel descending pathways of central origin (Fig. 6). The assumption of separate pathways for controlling the focal and postural components was necessitated by the known flexibility of their relative timing depending upon task conditions (Benvenuti et al. 1990; Horak and Nashner 1986; Lee et al. 1990; Zattara and Bouisset 1986). Based on the evidence that the onset of focal movement can be held back until the required APA is fully developed (Cordo and Nashner 1982), an inhibition on the control of movement from the process that controls postural support was also postulated.

Massion did not consider the case of MI, which involves a process that inhibits focal movement (Jeannerod 2006). Only recently has it been demonstrated that postural adjustments (Boulton and Mitra 2013, 2015; Grangeon et al. 2011; Rodrigues et al. 2010) and autonomic preparation (Collet et al. 2013) planned in support of imagined movement can escape this inhibition. Evidence for incomplete inhibition during MI is not confined to postural adjustments, but also includes observations of specific but attenuated EMG activity in muscles that would be activated if the movement was executed (Bonnet et al. 1997; Guillot et al. 2007; Lebon et al. 2008). Massion also did not elaborate the architecture in respect of anticipatory and compensatory components of posture control. A key purpose of the present study and Wider et al. (2020) was to ascertain whether postural movements that accompany MI do have an anticipatory component. The possibility of this was clearly indicated by Boulton and Mitra’s (2015) finding that postural movements during periods of MI are sensitive to imagined constraints on the movements being imaged. This suggested that the postural activity that was not being fully inhibited during MI was of central origin as it could incorporate task-specific cognitive constraints. The present study and Wider et al. (2020) have shown not only that postural movement during manual MI has an anticipatory component, but also that CPM following MI may or may not be preceded by APM before MI onset (as was the case for O in the externally triggered conditions). This pattern of findings reinforces the necessity of expanding the control architecture to explicitly address both MI and anticipatory and compensatory postural components.

**Fig. 7:**
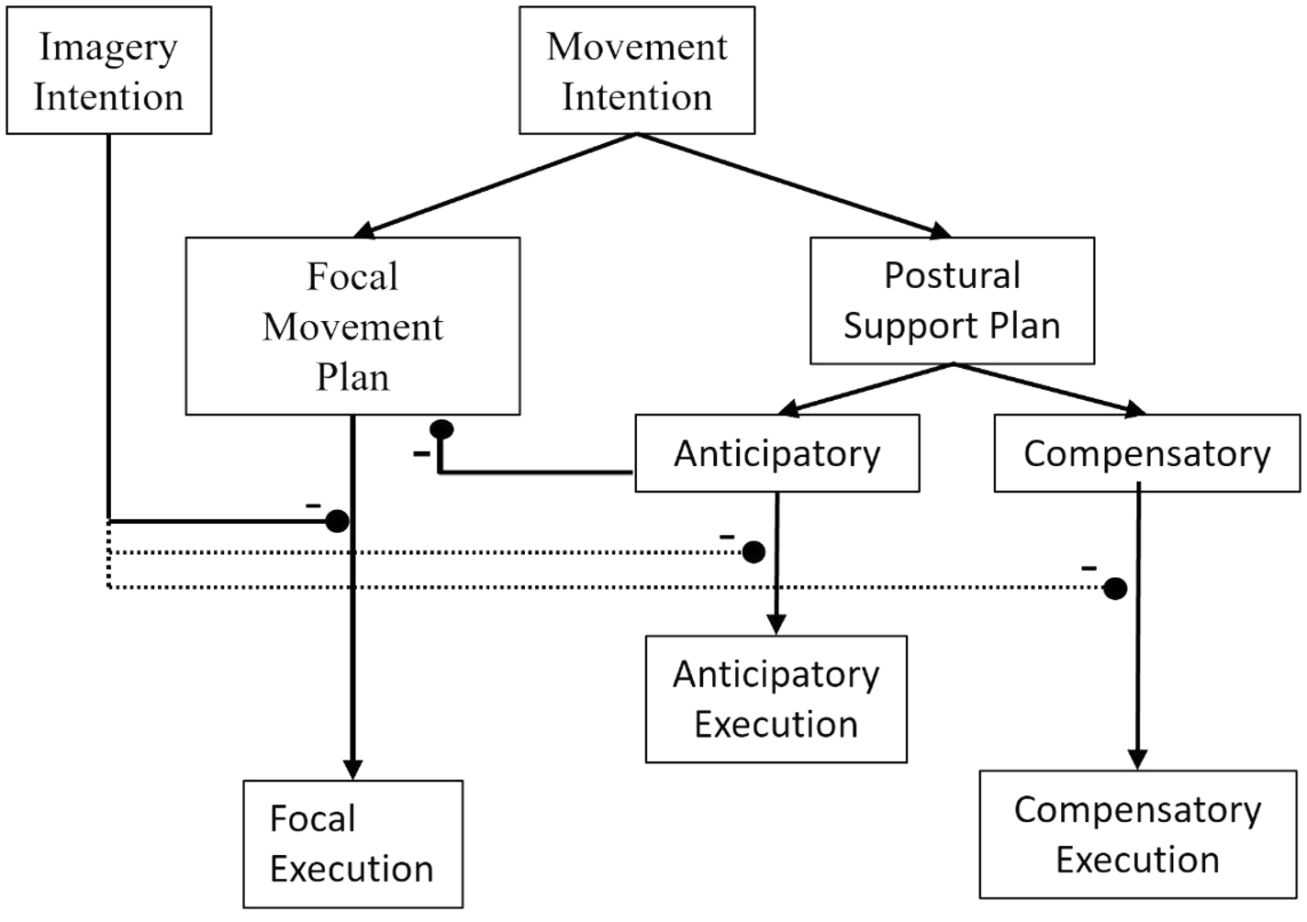
Proposed control architecture for focal movement and postural support during movement execution and MI

We have schematised our proposed architecture in Fig. 7. The proposed architecture separates control pathways for the anticipatory and compensatory elements of the postural control (Aruin et al. 2001). Leaving aside the actions associated with imagery intention for the time being, the movement intention aspect proposes parallel focal movement and postural support plans of central origin (as did Massion, Fig. 6). We represent the anticipatory and compensatory components of the postural support plan as parallel processes. The focal movement and compensatory postural support are tightly linked and co-occur in the case of movement execution. The anticipatory component may or may not occur depending upon its necessity and the ability to plan it. Where movement onset is externally triggered, for example, there may not be enough time or information to take anticipatory action. Previous and present results on movement execution, and present results on MI, suggest that old age brings with it a specific deficit in generating the anticipatory postural component when the focal movement’s timing must coordinate with an unpredictable external cue. Note that an inhibition pathway is proposed from the anticipatory arm of the postural support plan to the focal movement plan. This is the analogue here of the inhibition depicted in Fig. 6, proposed to accommodate observations in the literature that the timing of focal movements can be modulated based on the time requirements of anticipatory postural adjustments (e.g., Cordo and Nashner 1982).

Next, we consider the case of MI, which we have depicted as the imagery intention process. This view of what occurs during MI is based on the proposal that MI involves an inhibition process that counteracts the focal movement commands before they activate peripheral effectors (Collet and Guillot 2009; Jeannerod 2006). Any such inhibition is understood to be incomplete, as it does not eliminate autonomic arousal, EMG activity in involved muscles, or the postural adjustments accompanying motor planning (Collet et al. 2013; De Souza et al. 2015; Guillot et al. 2007). Accordingly, an inhibitory influence from imagery intention to the focal movement plan is indicated in Fig. 7. This inhibition appears as a solid line as, in many instances, focal movement can be completely absent during MI. Inhibitory influences are also indicated from imagery intention to the anticipatory and compensatory components of the postural support plan, but these are dashed lines to indicate that these pathways do not achieve complete attenuation of postural adjustments, as has been shown in the present experiments and Boulton and Mitra (2013, 2015) and Mitra et al. (2016). Aside, from enabling insights into the postural component of focal movement planning (without contamination from execution processes) the discovery of incomplete inhibition of postural adjustments during MI presents potential practical benefits in training and rehabilitation.

As we have noted already, the absence of APM preceding O’s executed and imagined arm movement in the externally triggered condition has potentially important practical consequences for active and independent living. Limb movements that must be coordinated with environmental events of unpredictable timing are an everyday necessity in navigating civic spaces and interacting socially. Raising the arm while standing upright does not even include the variable spatial constraints that are often added to the temporal uncertainties of coordinating with external events. Take, for example, the active destabilization of body posture that occurs when the trunk must bend as part of the focal movement, resulting in a large change in CG position (e.g., in Stapley et al. 1998). Previous research on postural support for physical movements has shown that O produce weaker and delayed APA (Inglin and Woollacott 1988; Man’kovskii et al. 1980; Rogers et al. 1992; Woollacott and Manchester 1993), and, as a result, larger CPA that can have destabilizing effects (Kanekar and Aruin (2014a). In the present results, O’s absence of APM for physical arm movements and MI in the externally triggered condition suggests that the issue occurs at the level of planning the postural support for the movement that is to be coordinated with external events. Note that there are CPMs without preceding APMs when there is no focal movement. Thus, the CPM is not counteracting a perturbation in the direction opposite to it. Such CPMs could destabilise the body if they are large in magnitude.

The clear identification of APM preceding MI and the sensitivity of these APMs to task conditions suggests that MI training could be used to stimulate postural anticipation. For example, adding a load to the arm being raised increases the magnitude of postural anticipation that is required. Similarly, a manual action like catching an object adds load to the body and elicits anticipatory postural activity (Scariot et al. 2016). Training O’s with the history of falls in such catching (and throwing) actions has shown promise in improving balance confidence (Arghavani et al. 2019). If MI of such activities also improves balance performance and confidence, particularly the anticipatory component, training interventions could be extended to individuals for whom repeatedly performing such actions may not be safe or possible.

## Abbreviations

AP: Anteroposterior
APA: Anticipatory postural adjustment
APM: Anticipatory postural motion
CG: Centre of gravity
CPA: Compensatory postural adjustment
CPM: Compensatory postural motion
EPA: Early postural adjustment
MI: Motor Imagery
ML: Mediolateral
O: Old
Y: Young
